# Epitope Coverage of Anti-SARS-CoV-2 Nucleocapsid IgA and IgG Antibodies Correlates with Protection against Re-Infection by New Variants in Subsequent Waves of the COVID-19 Pandemic

**DOI:** 10.3390/v15020584

**Published:** 2023-02-20

**Authors:** Michelle O. Mullins, Muneerah Smith, Hazel Maboreke, Andrew J. M. Nel, Ntobeko A. B. Ntusi, Wendy A. Burgers, Jonathan M. Blackburn

**Affiliations:** 1 Department of Integrative Biomedical Sciences, Faculty of Health Sciences, University of Cape Town, Cape Town 7925, South Africa; 2 Department of Medicine, University of Cape Town and Groote Schuur Hospital, Cape Town 7925, South Africa; 3 Wellcome Centre for Infectious Diseases Research in Africa, University of Cape Town, Cape Town 7925, South Africa; 4 Division of Medical Virology, Department of Pathology, University of Cape Town, Cape Town 7925, South Africa; 5 Institute of Infectious Disease and Molecular Medicine, Faculty of Health Sciences, University of Cape Town, Cape Town 7925, South Africa

**Keywords:** immunoassay, epitope coverage, quantitative antibody binding, protein microarray, SARS-CoV-2 antibodies, humoral response, SARS-CoV-2 re-infection

## Abstract

The COVID-19 pandemic continues to affect individuals across the globe, with some individuals experiencing more severe disease than others. The relatively high frequency of re-infections and breakthrough infections observed with SARS-CoV-2 highlights the importance of extending our understanding of immunity to COVID-19. Here, we aim to shed light on the importance of antibody titres and epitope utilization in protection from re-infection. Health care workers are highly exposed to SARS-CoV-2 and are therefore also more likely to become re-infected. We utilized quantitative, multi-antigen, multi-epitope SARS-CoV-2 protein microarrays to measure IgG and IgA titres against various domains of the nucleocapsid and spike proteins. Potential re-infections in a large, diverse health care worker cohort (N = 300) during the second wave of the pandemic were identified by assessing the IgG anti-N titres before and after the second wave. We assessed epitope coverage and antibody titres between the ‘single infection’ and ‘re-infection’ groups. Clear differences were observed in the breadth of the anti-N response before the second wave, with the epitope coverage for both IgG (*p* = 0.019) and IgA (*p* = 0.015) being significantly increased in those who did not become re-infected compared to those who did. Additionally, the IgG anti-N (*p* = 0.004) and anti-S titres (*p* = 0.018) were significantly higher in those not re-infected. These results highlight the importance of the breadth of elicited antibody epitope coverage following natural infection in protection from re-infection and disease in the COVID-19 pandemic.

## 1. Introduction

Despite extensive research, the development and roll-out of vaccines to protect against COVID-19 disease, SARS-CoV-2 infections, re-infections, and breakthroughs has continued across the globe—albeit with the pandemic taking apparently different courses in different geographic regions. As part of efforts to curb the pandemic, it is important to gain a more complete understanding of the role of both the innate and adaptive immune responses in COVID-19 disease. COVID-19 presents as a spectrum of disease, with some individuals experiencing more severe symptoms and a worse disease prognosis than others [1,2,3], suggesting that host factors play a key role in determining the outcome of infection. Amongst others, the humoral immune response between highly exposed individuals differs drastically in terms of titres, epitope utilization, functionality, and longevity. 

Furthermore, it is evident that previously infected individuals are becoming re-infected [4,5,6,7,8,9,10], suggesting: (1) a suboptimal immune response; (2) that some individuals are not mounting an immune response; (3) waning levels and decreased avidity [11,12,13] of antibodies post-infection—as previously seen for SARS-CoV-1 [14] and MERS-CoV [15]—or (4) that the immune response is not protective across all the SARS-CoV-2 strains that have emerged. Identifying immunoglobulins that correlate with protection from infection or re-infection and disease may assist us in understanding the mechanisms behind an optimal immune response; this may also shed additional light on the reasons behind varying disease prognoses.

Many mono-epitope, qualitative antibody tests are available; however, these cannot provide detailed information regarding immune targets, how these vary between individuals and over time, and the functionality of the different immunoglobulins. Here, we used a high-throughput, quantitative SARS-CoV-2 multi-antigen, multi-epitope immunoassay to shed light on differential antibody responses in individuals who became re-infected in subsequent waves of the COVID-19 pandemic, compared to those who did not. This assay utilizes KREX protein folding technology, which uses biotin carboxyl carrier protein (BCCP) as a marker for correct folding and to immobilize the individual domains of the recombinant SARS-CoV-2 nucleocapsid and spike antigens [2,16]. 

Healthcare workers (HCW) are assumed to be exposed to SARS-CoV-2 at a higher rate than the general population and are therefore considered to be at a higher risk of contracting COVID-19. Due to this increased exposure, it is also likely that these individuals represent an enriched cohort to study re-infection. Here, we aimed to determine the rate of infection and re-infection amongst a South African HCW cohort and to use this information to identify IgG and IgA correlates of protection against re-infection.

## 2. Materials and Methods

### 2.1. Cohort Information and Sample Collection 

A prospective observational healthcare worker cohort was used for this study. The clinical characteristics of this cohort is summarized in Table 1. 

**Figure 1:**
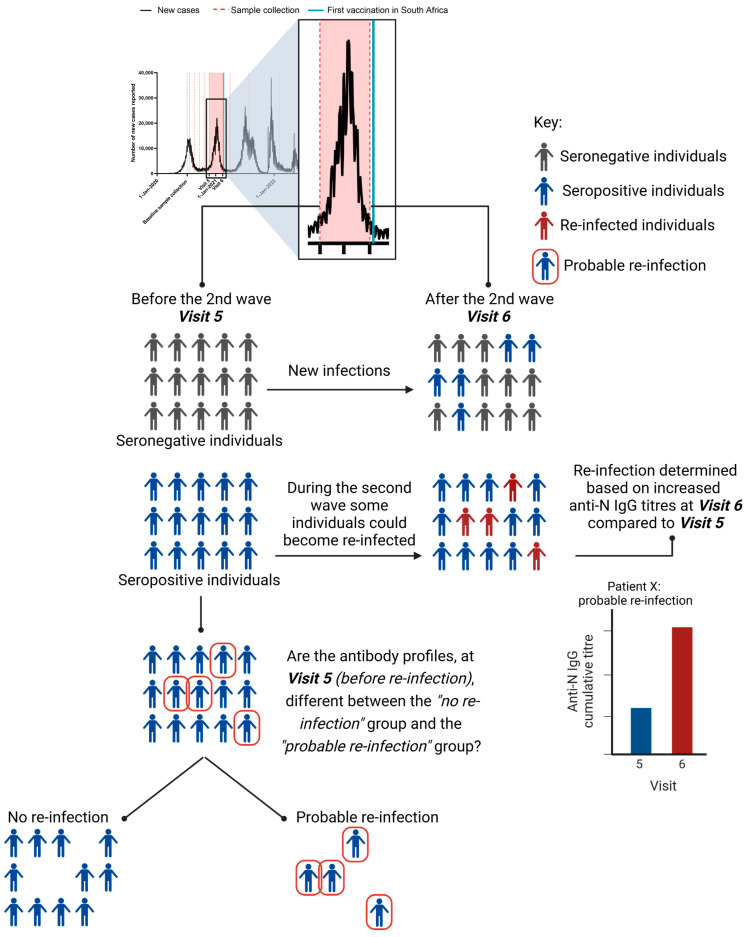
Study design. Number of COVID-19 cases in South Africa between January 2020 and November 2022. Red dotted lines represent dates of sample collection from HCW cohort. The red-shaded block represents the time period assessed in this study. The blue line represents start of the vaccine rollout in South Africa. (Data adapted from WHO [17]). The schematic below represents the study design and how probable re-infections were identified. Created with BioRender.com (accessed on 15 February 2023).

A total of 300 participants were enrolled from Groote Schuur, Victoria, and Somerset Hospitals. Blood samples were collected longitudinally from the enrolled participants. The Sisonke vaccine rollout of the Ad26.COV2.S vaccine to HCWs began in South Africa on 17 February 2021, which was post-Visit 6 (Figure 1), and all participants were non-vaccinated at the time-points analysed here.

Plasma was isolated from samples by centrifugation and aliquots stored at −80 °C until needed. Ethics approval (HERC Ref 210/2020) for this study was obtained from the Human Research Ethics Committee of the Faculty of Health Sciences, University of Cape Town, South Africa. Full consent was obtained from all participants prior to sample collection. 

Fifty pre-pandemic HIV positive serum samples were used as true negative controls. In this cohort, no additional clinical annotations were provided.

### 2.2. Protein Microarrays

SARS-CoV-2 multi-antigen, multi-epitope arrays containing component-resolved nucleocapsid (N) domains and intrinsically-disordered epitopes, as well as spike ectodomain trimer (S), S1, and RBD domains, were sourced commercially (ImmuSAFE array v4; Sengenics Corporation, Singapore) and were used as per the manufacturer’s instructions.

### 2.3. Serological Assays 

Microarray slides were blocked in blocking buffer (20% Glycerol, 25 mM HEPES buffer (pH 7.4), 50 mM KCl, 1% Triton X-100, 1 mM DTT and 50 µM Biotin) for 1 h and washed 2 × 5 min in PBST (PBS, 0.2% Tween-20, pH 7.4) and 2 × 5 min in PBS. The slides were dried by centrifugation at 1200× *g* at 23 °C for 2 min. Individual arrays were isolated using ProPlate 24 plex multi-well chambers (GraceBio-Labs, Bend, OR, USA). Prior to assays, plasma samples were incubated with 0.1% Triton X-100 for 1 h on ice to deactivate potential live virions, then diluted 1:50 in assay buffer (PBST, 1% BSA, 1% milk powder). Each microarray was incubated with plasma for 1 h at room temperature at 100 RPM. The wells were briefly rinsed with PBST, and then the slides were removed from the gaskets and washed 2 × 5 min in PBST and 2 × 5 min in PBS. The slides were dried by centrifugation at 1200× *g* at 23 °C for 2 min. Slides were then incubated with detection antibodies (10 µg/mL Cy3-labelled anti-human IgG and 10 µg/mL AF647-labelled anti-human IgA in assay buffer) for 30 min at RT at 100 RPM. The wells were briefly rinsed with PBST, and then the slides were removed from the gaskets and washed 2 × 5 min in PBST and 2 × 5 min in PBS. The slides were dried by centrifugation at 1200× *g* at 23 °C for 2 min. 

### 2.4. Bioinformatic Analysis

#### 2.4.1. Image Analysis: Raw Data Extraction and Data Pre-Processing 

Slides were scanned at a fixed gain setting using an InnoScan 710 (Innopsys, Carbonne, France) fluorescence microarray scanner, generating a 16-bit TIFF file. A visual quality control check was conducted, and any arrays showing spot merging or other artefacts were re-assayed. A GAL (GenePix Array List) file containing information regarding the location and identity of all probed spots was used to aid with image analysis. Automatic extraction and quantification of each spot were performed using Mapix software version 8.1.1 (Innopsys, Occitanie, France), yielding the median foreground and local background pixel intensities for each spot. 

The mean net fluorescence intensity of each spot was calculated as the difference between the raw mean intensity and its local background. Extrapolated data were filtered and normalized using an in-house-developed software (CT100+ programme). Human IgG and IgA (detected by fluorescently labelled secondary antibody) and human anti-IgG (detected only when plasma or serum is added to the slide) were used as positive controls to assess image signal intensity.

Reciprocal titres per-antigen were determined from the measured net fluorescence intensity, based on the projected further dilution of the sample required to reach the limit of detection in the assay, according to the following equation:

(1) $$\mathrm{Reciprocal}\;\mathrm{Titer}=\frac{\mathrm{Net}\;\mathrm{intensity}\;\left(\mathrm{RFU}\right)\times \mathrm{initial}\;\mathrm{serum}\;\mathrm{dilution}}{\mathrm{Limit}\;\mathrm{of}\;\mathrm{detection}\;\left(\mathrm{RFU}\right)}$$

#### 2.4.2. Statistical Tests 

Statistical analyses and graphical representations were generated using GraphPad Prism (v 9.0; GraphPad Software, San Diego, CA, USA). A one-way ANOVA with Welch’s correction was applied to determine the statistical significance of the differences observed between independent groups.

## 3. Results

### 3.1. Seroprevelance 

The N protein is generally favoured over the S protein for seroprevalence studies due to the faster rates of clearance of the anti-S response post-infection [18]. We therefore used the anti-N IgG titre to assess seropositivity in our cohort across three different timepoints (Table 2) representing early in the first wave, prior to the onset of the second wave, and post-second wave infections in South Africa. The ImmuSAFE microarray platform has previously been reported to have 100% sensitivity and specificity in detecting anti-N antibody responses to SARS-CoV-2 [2]. 

### 3.2. Antibody Profiles Post Potential Re-Infection 

Due to new strains and sub-optimal immune responses, it is likely that individuals who are highly exposed to SARS-CoV-2 may become re-infected. Due to the relative stability of the anti-N IgG titres over time, observed here and elsewhere [18], it is possible that an increase in anti-N IgG titres > 1 month after a previous infection could be indicative of re-infection. The opposite is true for those who do not become re-infected; we would expect to see a decrease or no change in the anti-N IgG titres. 

Therefore, we assessed antibody titres against the nucleocapsid protein on the ImmuSAFE microarrays, as described previously [2], at Baseline (July/August 2020, early- mid-wave 1; driven by ancestral SARS-CoV-2), Visit 5 (November 2020, before the second wave) and Visit 6 (January 2021; late in the second wave, which was driven by the β SARS-CoV-2 variant) to identify individuals who likely became re-infected during the second wave (Figure 1). It is important to note that the individuals who were seropositive at Visit 5 were not all infected at the same time and were not all seropositive at baseline. 

Individuals who were seropositive prior to the second wave were selected and their anti-N titres were assessed at subsequent timepoints to identify individuals who showed an increase in antibody titres late in the second wave. The influence of BMI, sex, age, and ethnicity on re-infection status was assessed, however no significant differences were observed (Figure S1).

**Figure 2:**
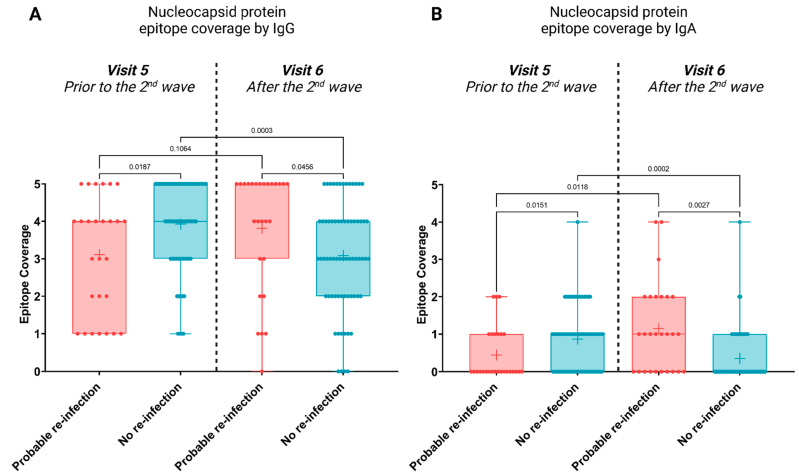
N-protein epitope coverage by IgG and IgA-specific antibodies in seropositive individuals before and after the second COVID-19 wave in South Africa (Visit 5 and 6). Participants were split into two groups based on potential re-infections. (**A**) IgG N-protein epitope coverage. (**B**) IgA N-protein epitope coverage. Pairwise comparisons were made using a one-way ANOVA, and *p*-values were calculated using Welch’s correction to compare the mean of each category to the mean of each other category. Samples sizes: probable re-infection: n = 28, no re-infection: n = 59.

**Figure 3:**
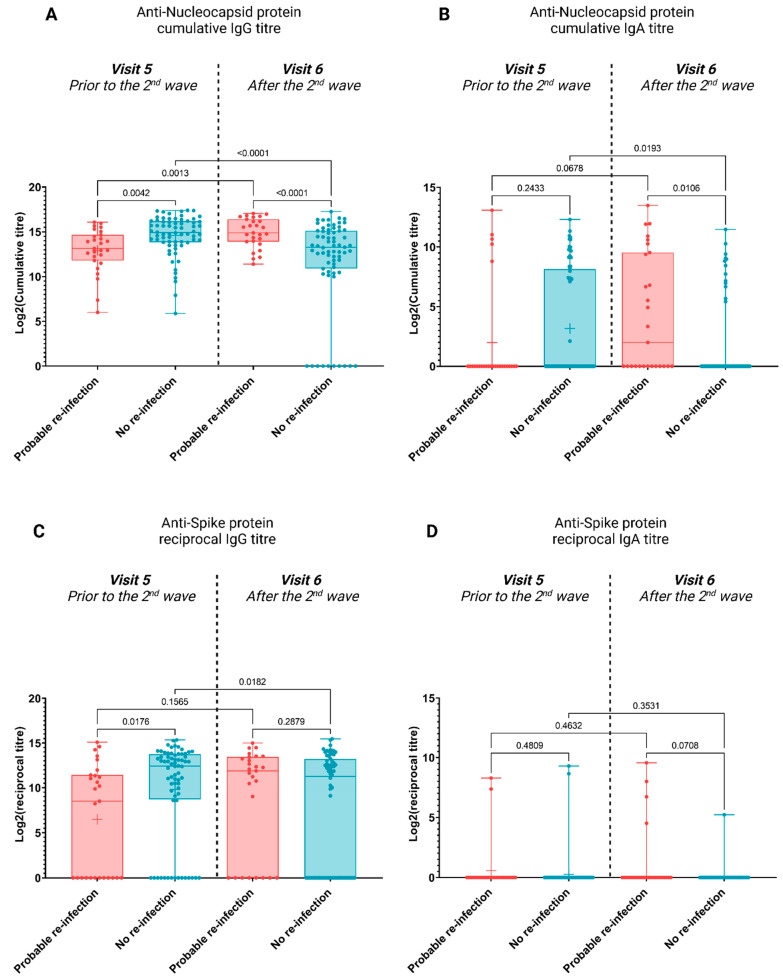
Anti-N and anti-S IgA and IgG titres in seropositive individuals before and after the second COVID-19 wave in South Africa (Visit 5 and 6). Participants were split into two groups based on potential re-infections. (**A**) Anti-N IgG cumulative reciprocal titre. (**B**) Anti-N IgA cumulative reciprocal titre. (**C**) Anti-S IgG reciprocal titre. (**D**) Anti-S IgA reciprocal titre. Pairwise comparisons were made using a one-way ANOVA, and *p*-values were calculated using Welch’s correction to compare the mean of each category to the mean of each other category. Samples sizes: probable re-infection: n = 28, no re-infection: n = 59.

An epitope coverage score was calculated as the sum of the number of IgG or IgA-positive N-protein epitopes (C- and N-terminal domains, and three intrinsically disordered regions) for each sample. This score represents the breadth of the antibody response (2). The epitope utilisation and antibody titres of the participants who appear to have become re-infected (n = 28) were then compared to those who were seropositive but did not become re-infected (n = 59; Figure 2 and Figure 3). 

For both IgG and IgA responses, a significantly broader epitope coverage was observed at Visit 5—prior to re-exposure—in individuals who did not become re-infected during the second wave (Figure 2A: *p*-value = 0.0187, Figure 2B: *p*-value = 0.0151); thus, epitope utilisation appears to correlate with protection from re-infection. Following re-infection (Visit 6), the IgG and IgA epitope utilisation broadened, compared to before (Figure 2A,B, respectively). Furthermore, re-infection resulted in a significantly broader epitope coverage, for both IgG and IgA, compared to those who were not re-infected (Figure 2A: *p*-value = 0.0456, Figure 2B: *p*-value = 0.0027)—as expected. 

The relationship between antibody titres and re-infection was assessed. For both N- and S-protein, IgG titres were significantly higher in non-re-infected individuals prior to the second wave (Figure 3A,C, *p*-value = 0.0042 and *p*-value = 0.0176, respectively). However, no statistically significant differences were observed for IgA titres prior to the second wave (Figure 3B,D, *p*-value = 0.2433 and *p*-value = 0.4809, respectively). Following the second wave, those with inferred re-infection showed significant increases in IgG anti-N titres (Figure 3A, *p*-value = 0.0013), and whilst an increase in IgG anti-S titres was observed, this was not significant (Figure 3C). An increase in IgA anti-N and anti-S titres was observed; however, this result was not significant (Figure 3B,D, respectively). 

## 4. Discussion

As we face the ongoing challenges of the COVID-19 pandemic, the global interest in understanding the role of T- and B-cell responses in protection from (re)-infection remains a priority. Despite high seroprevalence and vaccination rates, re-infections and breakthrough infections are continually reported [4,5,6,7,8,9,10]. Multiple factors could contribute to this incomplete protection from infection and disease. Serological assays can be used to determine the magnitude, breadth, and durability of the humoral immune response against SARS-CoV-2 and how these factors influence the rates of (re)-infection. Many studies have reported waning antibody levels post-infection and vaccination, with the duration of titres above the detectable level ranging from 5–8 months [19,20]. Furthermore, the effects of age, ethnicity, sex, and disease severity on infection rates, antibody titres, and epitope coverage have been studied in cross-sectional cohorts [2,21,22]; however, the effects of these factors on the durability of protection remains largely unknown. 

With the emergence of new SARS-CoV-2 variants, coupled with waning and incomplete immune responses, the rise in cases of re-infection is not surprising. There is a need, therefore, for tools to predict whether natural- or vaccine-induced immunity offers protection against infection and/or disease. 

The first wave in South Africa was driven by the ancestral SARS-CoV-2 virus, whereas the second wave was largely driven by the Beta variant (B.1.351). Here, we have assessed anti-nucleocapsid and anti-spike protein IgG and IgA responses in the plasma of HCWs before and after the second COVID-19 wave to identify probable re-infections. We and others have observed that the anti-N antibody titres in previously SARS-CoV-2-infected individuals are relatively long-lived, but routinely decline as a function of time in the absence of any re-infection [18]. Since the sequence homology between the SARS-CoV-2 and endemic hCoV nucleocapsid proteins is relatively low, and since cross-reactivity with the anti-hCoV-N antibodies has been engineered out in the ImmuSAFE arrays used here [2], this suggests that the increase in anti-N titres observed following the second wave of infections in South Africa is strongly indicative of re-infection. 

The antibody profiles of those re-infected and those not, were compared to identify a candidate correlate of protection. Our data on the breadth and magnitude of the antibody responses (Figure 2 and Figure 3) clearly shows an importance for both the magnitude and breadth of the humoral immune response to elicit protection from re-infection with new SARS-CoV-2 strains. 

It is possible to assess the patterns of protection from re-infection as SARS-CoV-2 infections came in punctuated waves of infections, with new strains driving each wave. However, influenza, for example, has the added factor that most individuals are likely to have been exposed multiple times. Vaccinations and natural influenza infections are thought not to induce broadly neutralizing antibodies that protect against different influenza strains [23]; however, the breadth of anti-influenza antibody responses is reported to be greater following natural infection in comparison to vaccination, including eliciting differing immunodominance patterns [24,25]. 

Immunodominance could lead to pathogen escape via mutation; therefore, it seems immunologically plausible that if multiple different epitopes on viral antigens are targeted by antibodies (including non-neutralizing antibodies with Fc effector functions), then the subsequent variant strains may be less able to escape all the antibodies present. Neutralization-related epitopes provide an incomplete picture of the role of antibodies in providing protection against infection. Here, we used the epitope coverage of anti-N antibody responses as a proxy of the breadth of the anti-SARS-CoV-2 antibody response and to observe a statistically-significant correlation with protection against re-infection (Figure 2). 

It is important to note that in this study we measured anti-N epitope coverage using serum antibodies, whereas protection against (re)-infection by SARS-CoV-2 seems likely to manifest through tissue resident B-cell-derived anti-S sIgA antibodies in mucosal tissues, rather than through bone marrow-derived monomeric IgG or IgA in blood. It remains to be determined, therefore, whether the breadth of anti-N epitope coverage by serum antibodies is reflected by anti-S epitope coverage by sIgA in mucosa, or whether there is some other immunological explanation (e.g., the breadth of the anti-SARS-CoV-2 T-cell responses) for the correlation between anti-N epitope coverage, titre, and re-infection observed here. Further research on mucosal samples from this cohort is now underway to investigate this.

### Limitations

While this study is statistically powered to identify longitudinal changes in antibody titres and epitope utilization, the ability to determine the influence of age, ethnicity, disease severity, and sex on the chances of re-infection is not possible due to the cohort size and the number of participants we inferred were re-infected (Figure S1). Additionally, whilst we have assumed re-infection in some individuals, this was not clinically proven, nor was the re-infecting virus sequenced to determine the variant.

In individuals who did not become re-infected, we cannot prove re-exposure. However, given the increased rate of seropositivity observed at Visit 5 (Table 2), it seems reasonable to infer ongoing exposure in a significant proportion of the previously-infected-but-not-re-infected individuals in our cohort. Furthermore, if absence of re-infection was determined entirely by non-pharmaceutical interventions rather than by immunological factors, then it would seem unlikely that a statistically significant correlation between epitope coverage, antibody titre, and re-infection would have been observed by chance. Importantly, since all Visit 5 samples were collected prior to the Sisonke rollout of the Ad26.COV2.S vaccine to HCWs in South Africa and, moreover, since all vaccines trialled in South Africa prior to Visit 5 (principally the ChAdOx1, BNT162b2, and Ad26.COV2.S vaccines) would have elicited pure anti-S antibody responses, it seems reasonable to assume that vaccination status was not a confounding variable in this study.

## Figures and Tables

**Table 1:** Characteristics of the COVID-19 HCW cohort.

Characteristic		
Gender	Female	220
	Male	77
	Not declared	3
Age distribution	18–30	89
	31–40	80
	41–60	105
	61–73	5
	Not declared	21
Ethnicity	African	107
	Caucasian	69
	Coloured	99
	Asian	16
	Other	3
	Not declared	6

**Table 2:** Seroprevalence based on anti-N IgG titres. Sample size: HCW: n = 300. nd = not determined.

Sampling Time	Baseline	Visit 5	Visit 6
Seroprevalence (%)	30.67	44.92	61.97
PCR+ (%)	14.00	nd	nd

## Data Availability

Data are contained within the article.

## Supplementary Materials

The following supporting information can be downloaded at: https://www.mdpi.com/article/10.3390/v15020584/s1, Figure S1: Influence of BMI, age, sex, and ethnicity on the likelihood of re-infection.
