# Supplementary material for: Epitope Coverage of Anti-SARS-CoV-2 Nucleocapsid IgA and IgG Antibodies Correlates with Protection against Re-Infection by New Variants in Subsequent Waves of the COVID-19 Pandemic
Source: Viruses. 2023 Feb 20;15(2):584. doi: 10.3390/v15020584 (PMC9967965; doi:10.3390/v15020584)
Supplement: Supplementary file 1 [file viruses-15-00584-s001.zip › viruses-2149269-supplementary.pdf]

Supplementary material

Epitope Coverage of Anti-SARS-CoV-2 Nucleocapsid IgA and IgG Antibodies Correlates with Protection against Re-Infection by New Variants in Subsequent Waves of the COVID-19 Pandemic

Michelle O. Mullins [1], Muneerah Smith [1], Hazel Maboreke [1], Andrew J. M. Nel [1], Ntobeko A. B. Ntusi [2], Wendy A. Burgers [3,4,5] and Jonathan M. Blackburn [1,5,*]

1 Department of Integrative Biomedical Sciences, Faculty of Health Sciences, University of Cape Town, Cape Town 7925, South Africa
2 Department of Medicine, University of Cape Town and Groote Schuur Hospital, Cape Town 7925, South Africa
3 Wellcome Centre for Infectious Diseases Research in Africa, University of Cape Town, Cape Town 7925, South Africa
4 Division of Medical Virology, Department of Pathology, University of Cape Town, Cape Town 7925, South Africa
5 Institute of Infectious Disease and Molecular Medicine, Faculty of Health Sciences, University of Cape Town, Cape Town 7925, South Africa
* Correspondence: jonathan.blackburn@uct.ac.za; Tel.: +27-214-066-071

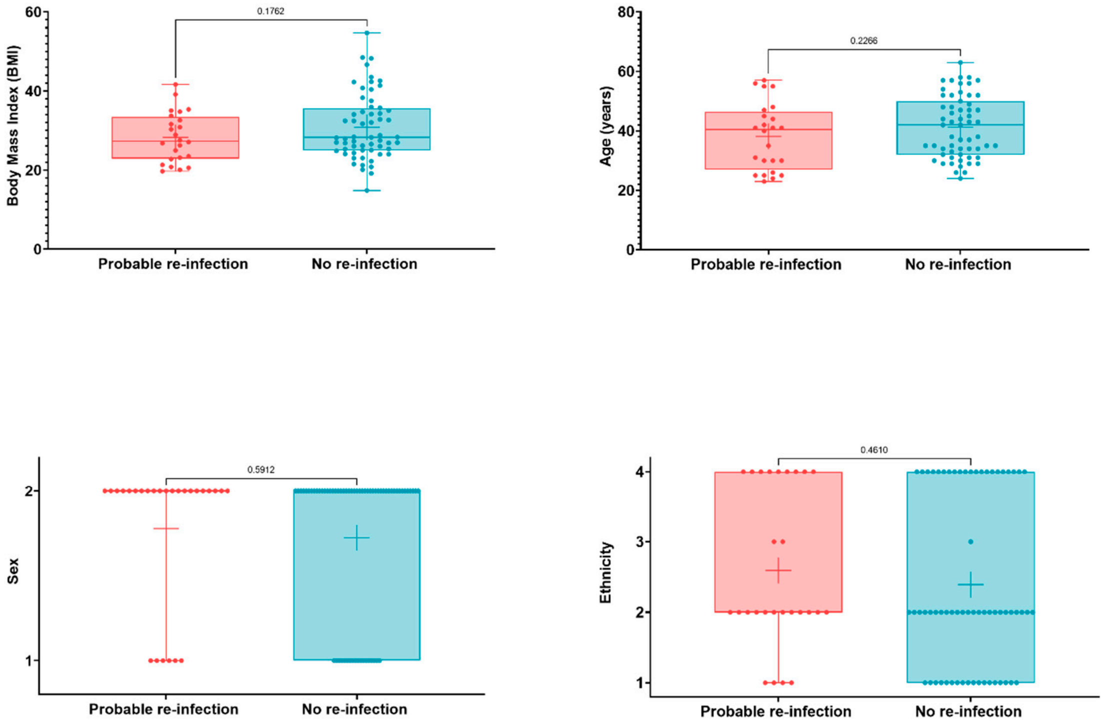

Figure S1: Influence of BMI, age, sex, and ethnicity on the likelihood of re-infection. Sex: 1 = male, 2 = female; Ethnicity: 1 = white, 2 = mixed ethnic groups, 3 = Asian, 4 = black/African. Pairwise comparisons were made using a one-way ANOVA, and p-values were calculated using Welch's correction to compare the mean of each category to the mean of each other category. Samples sizes: probable re-infection: n = 28, no re-infection: n = 59.
